# A New Approach for the Prevention and Treatment of Cardiovascular Disorders. Molecular Hydrogen Significantly Reduces the Effects of Oxidative Stress

**DOI:** 10.3390/molecules24112076

**Published:** 2019-05-31

**Authors:** Tyler W. LeBaron, Branislav Kura, Barbora Kalocayova, Narcis Tribulova, Jan Slezak

**Affiliations:** 1 Centre of Experimental Medicine, Institute for Heart Research, Slovak Academy of Sciences, Bratislava 841 04, Slovak Republic; LeBaronT@molecularhydrogeninstitute.com (T.W.L.); branislav.kura@savba.sk (B.K.); barbora.kalocayova@savba.sk (B.K.); narcisa.tribulova@savba.sk (N.T.); 2 Molecular Hydrogen Institute, Enoch City, UT, 847 21, USA

**Keywords:** heart transplantation, ischemia/reperfusion injury, molecular hydrogen, oxidative stress, radiation-induced heart disease

## Abstract

Cardiovascular diseases are the most common causes of morbidity and mortality worldwide. Redox dysregulation and a dyshomeostasis of inflammation arise from, and result in, cellular aberrations and pathological conditions, which lead to cardiovascular diseases. Despite years of intensive research, there is still no safe and effective method for their prevention and treatment. Recently, molecular hydrogen has been investigated in preclinical and clinical studies on various diseases associated with oxidative and inflammatory stress such as radiation-induced heart disease, ischemia-reperfusion injury, myocardial and brain infarction, storage of the heart, heart transplantation, etc. Hydrogen is primarily administered via inhalation, drinking hydrogen-rich water, or injection of hydrogen-rich saline. It favorably modulates signal transduction and gene expression resulting in suppression of proinflammatory cytokines, excess ROS production, and in the activation of the Nrf2 antioxidant transcription factor. Although H_2_ appears to be an important biological molecule with anti-oxidant, anti-inflammatory, and anti-apoptotic effects, the exact mechanisms of action remain elusive. There is no reported clinical toxicity; however, some data suggests that H_2_ has a mild hormetic-like effect, which likely mediate some of its benefits. The mechanistic data, coupled with the pre-clinical and clinical studies, suggest that H_2_ may be useful for ROS/inflammation-induced cardiotoxicity and other conditions.

## 1. Introduction

According to statistics cardiovascular and oncological diseases are the main cause of more than 93% of morbidity and mortality worldwide [1,2]. One of the most widely used methods for treating patients with oncological diseases is radiotherapy, which uses ionizing radiation. This treatment damages cancer cells leading to their apoptosis and eventual healing of patients [3]. However, during irradiation of cancer cells, the surrounding healthy tissue may also be inadvertently affected which, in turn may cause serious health complications, including radiation-induced heart disease [4,5]. Ischemia/reperfusion (I/R) injury represents a condition in which tissues or organs are damaged due to being exposed to a period of ischemia followed by replenishment of oxygen-rich blood. I/R injury is implicated in the pathogenesis of various clinical issues including stroke, myocardial infarction, organ transplantation, and also in injuries to various organs such as the brain, heart, kidneys and skeletal muscles [6]. Therefore, research in this field, and the use of completely new techniques that will positively influence the effects of excessive free radical production on the cardiovascular system, can significantly improve the quality of life of both oncology and cardiology patients. 

Molecular hydrogen (H_2_) represents an effective and non-toxic molecule with wide potential for treating many reactive oxygen/nitrogen species (ROS/RNS)-related diseases, including diseases induced by irradiation [7,8]. Besides the antioxidant action, hydrogen also exerts its beneficial effects through reduction of inflammation and modulation of signaling pathways, thus providing cytoprotection [9]. The protective effects of H_2_ have been investigated in pathological conditions such as cardiac fibrosis, hepatic injuries, neuronal diseases, radiation-induced diseases, diabetes, etc. [10], in which free radicals are casually involved. Ischemia and subsequent reperfusion of the heart represents another state in which an enormous number of tissue-damaging free radicals are produced. I/R could be an important situation in which to use H_2_, helping to mitigate/alleviate the negative impact of toxic ROS/RNS [6]. The great advantage of using H_2_ is also the wide spectrum of administration possibilities available to the organism – by inhalation, drinking H_2_-rich water produced with pressure or using H_2_-producing tablets, using H_2_-rich saline solution, or taking an H_2_ bath [8,11].

This article summarizes the most recently published literature about using H_2_ in different ROS/RNS-related diseases such as I/R injury, radiation-induced heart disease, and the potential using of H_2_ in transplantations and in grafts storage, which is closely connected with I/R injury. We also briefly discuss some of the molecular mechanisms of H_2_ and the viable potential for H_2_ in clinical situations.

## 2. ROS and Cardiovascular Diseases

**Figure 1:**
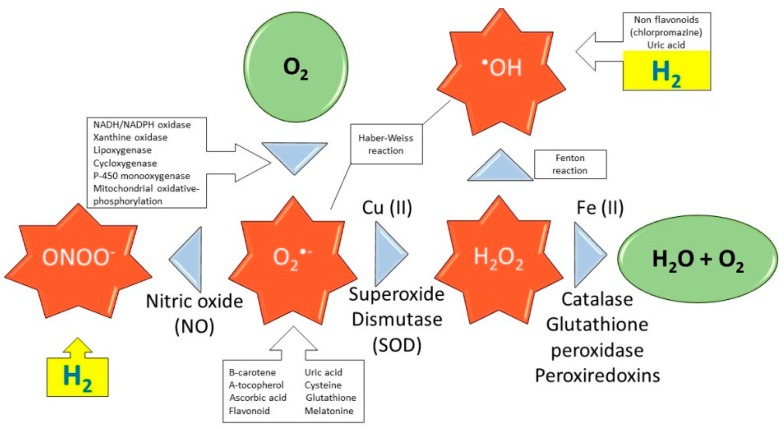
Production of ROS: effect of antioxidants and selective action of H_2_. Schematic reactions of ROS production by action of enzymes during respiration in mitochondria. Green color represents non-radical molecules, red color represents ROS created from normal respiration, yellow color indicates action of H_2_.

Oxidative stress occurs as a result of a dyshomeostasis between ROS/RNS and the antioxidant self-defense system. This dysregulation is considered a causative common denominator for many pathological processes, as it impairs cellular and organ function [12]. ROS are byproducts of oxygen reduction, which occurs during normal cellular metabolism. These ROS/RNS include superoxide anion (O_2_^•−^), hydroxyl (^•^OH), peroxyl (RO_2_^•^), alkoxyl (RO^•^) radicals, and nitric oxide (NO^•^), and other non-radical species, which can function as oxidizing agents, such as hydrogen peroxide (H_2_O_2_), hypochlorous acid (HOCl), ozone (O_3_), singlet oxygen (^1^O_2_), and peroxynitrite (ONOO^−^). The primary sources of ROS are mitochondrial respiration, NADH/NADPH oxidase, and xanthine oxidoreductase (Figure 1) [13]. Mitochondria are constantly exposed to high levels of ROS which, if unregulated, can cause mitochondrial DNA damage and cellular apoptosis [14].

ROS serve vital roles in modulating cell signaling pathways [15], gene expression, cell proliferation, apoptosis [16], DNA synthesis, cellular migration and invasion, tumor metastasis and angiogenesis [17,18]. Oxidative stress and/or nitrosative stress can activate several transcription factors, including nuclear factor (NF)-κB, activator protein 1, p53, hypoxia-inducible factor 1-α (HIF-1α), matrix metalloproteinases, peroxisome proliferator-activated receptor-γ, β-catenin/Wnt, and nuclear factor erythroid 2-related factor 2 (Nrf2) [19,20].

Hydroxyl and nitrosyl radicals, either by a direct reaction or by triggering a radical chain reaction, are the major contributors to the destruction of important biomolecules. Cellular redox dysregulation is one of the most important contributors to the pathogenesis of cardiovascular and metabolic diseases [21]. It has a causal role in various vascular diseases such as hypertension, diabetic vasculopathy, hypercholesterolemia and atherosclerosis. ROS mediate various signaling pathways that underlie cardiovascular pathophysiology [21]. Peroxynitrite plays a decisive role in the pathogenic mechanism for conditions such as stroke, myocardial infarction and chronic heart failure [22]. Indeed, acute and chronic excessive intracellular increases of ROS are implicated in the initiation and progression of cardiovascular diseases. For example, excessive ROS impair endothelial and vascular smooth muscle cell functions [23].

### 2.1. Radiation and Radiation-Induced Heart Disease

It has been demonstrated that a number of factors including intense exercise, cardiac infarction, cessation of blood flow, organ transplantation, inflammation, and radiation exposure can cause acute oxidative stress [24]. Radiation therapy is a primary method used to manage cancer; however, many noxious effects are inevitably linked to radiation exposure [25]. Radiation-induced symptoms are associated with increased ROS and inflammation during radiotherapy, which significantly impair the patient’s quality-of-life [26]. Exposure to a high dose of radiation over a short time is associated with acute radiation syndrome, whose symptoms are initially manifested as severe diarrhea and fluid loss [26]. 

The noxious consequences of radiation occur from either direct or indirect effects, which account for most of the damage [27]. Radiation induces a direct detrimental effect by directly damaging critical biomolecules including DNA, proteins, and lipids [28,29]. The indirect detrimental biological effect is attributed to toxic hydroxyl radicals (^•^OH) generated from radiolysis of H_2_O [9], which is estimated to account for 60–70% of the radiation-induced cellular damage. The hydroxyl radical is one of the most reactive ROS and reacts rapidly and indiscriminately with biological macromolecules. These biomolecules include DNA, which produces 8-hydroxy-2´-deoxyguanosine (8-OHdG, a biomarker of carcinogenesis) membrane lipids [30,31], as well as amino acids, and proteins. These reactions lead to the formation of various secondary ROS [32], which can result in severe health impairments due to cellular injury and irreversible damage in susceptible cells and organs [33]. Biomarkers such as malondialdehyde (MDA), thiobarbituric acid reactive substances (TBARs), etc. are indices of lipid peroxidation and membrane damage, which lead to pathological changes in membrane permeability [4,34,35,36]. 

Direct and indirect effects of radiation induce excessive production of eicosanoids (e.g., prostaglandins, prostacyclin, thromboxane and leukotrienes), which are endogenous mediators of inflammation, vasodilation or vasoconstriction, vascular permeability, and microthrombus formation [4]. Radiation injury to the myocardium is caused chiefly by these inflammatory aberrations in the microvasculature, leading to the activation of thrombin signaling and subsequent release of selectins and adhesion molecules [37]. This induces adhesion and extravasation of leukocyte transmigration of circulating monocytes resulting in vascular permeability and vasomotor changes in endothelial cells, which further induces leukocyte trafficking [38]. In the presence of elevated cholesterol, invading monocytes transform into activated macrophages and form fatty streaks in the intima [1]. Other pathological aberrations following irradiation include loss of alkaline phosphatase activity of capillary endothelial cells, and various pro-thrombotic changes such as production and release of von Willebrand factor (vWF), as well as a decreased production of thrombomodulin (Tm) and adenosine diphosphatase (ADPase) [39,40]. These changes further result in microthrombi, occlusion of vessels, reduced vascular density, perfusion defects, and focal ischemia, all of which lead to progressive myocardial cell death and fibrosis [1]. Cardiotoxic effects due to radiation exposure are collectively referred to as radiation-induced heart disease (RIHD).

Therefore, harmful effects of mediastinal irradiation include coronary artery disease, pericarditis, cardiomyopathy, valvular disease, conduction abnormalities [41], myocardial degeneration, perivascular and interstitial fibrosis [42], and chronic impairment of cardiac pump function [9,43]. Radiation-induced myocardial damage can occur 6-10 weeks after irradiation [42]. These noxious effects are related to dose, volume and technique of chest irradiation [1].

Cardiovascular injury due to radiation is the most common cause of adverse events among cancer survivors. Clinical studies of radiotherapy reveal regional perfusion defects in proportion to the radiation exposed area in non-symptomatic breast cancer patients [38]. Key factors responsible for the establishment of cardiovascular injury include oxidative stress, inflammation, and epigenetic modifications, and all have been linked to potential treatments [1,4,44]. Similar to the heart, the lungs are also very susceptible to radiation injury, which can result in radiation pneumonitis. Development of interstitial pneumonitis increases according to radiation dose, especially single-fraction total body irradiation at higher dose rates and higher total lung doses [45,46].

#### Methods of Radiation Protection

The increase in the prevalence of RIHD underscores the necessity to develop and procure new therapeutic methods to mitigate the noxious impact of radiation. The indirect effect of radiation exposure, i.e., high production of free radicals, is considered the primary mediator of radiation toxicity. Therefore, the blocking and scavenging of free radicals are the most important components in an effective protection strategy [47]. Highly effective radiation protectants with low toxicity are strongly desired and have always been emphasized in the field of radiation [48,49].

Free radical scavengers are able to act preventively and/or therapeutically. Many chemical substances can operate as free-radical scavengers to protect cells and tissues against oxidative damage. Research has identified many types of antioxidants including ascorbic acid, tocopherols, polyphenols, and thiols such as glutathione [50]. Some natural antioxidants such as vitamins, polyphenols, flavonoids, etc. often have fewer toxic effects but also provide lower radioprotection [4]. Therefore, an ideal radioprotectant should be one that is effective yet with few harmful side effects. Medical gases such as carbon monoxide (CO), hydrogen sulfide (H_2_S), and, as will be discussed later, molecular hydrogen (H_2_), may be used to attenuate the harmful effects of oxidative stress and radiation [51]. 

Irradiation induces upregulation of myocardial connexin-43 (Cx43), a decline of microRNA (miRNA)-1 and an increase of miRNA-21 levels [52]. This may be due to radiation-induced oxidative stress and/or inflammation, which result in these pathological changes and often accompany cardiovascular disease [53,54,55]. Treatment with aspirin and atorvastatin attenuates the irradiation-induced upregulation of myocardial Cx43 protein and miRNA-21 possibly via amelioration of oxidative stress and inflammation [56]. It also suppresses expansive remodeling by inhibition of macrophage infiltration [57]. The sulfhydryl compound amifostine is the only radioprotectant registered for use in humans, and has shown good radioprotective effects [58]. Unfortunately, it is not without significant side effects such as hypertension, nausea and vomiting, which understandably limits its clinical use [59].

### 2.2. Ischemia and Reperfusion Injury

Oxidative and inflammatory stress are also underlying causative factors in myocardial I/R injury. Cardiac myocytes, for their physiological function, require copious amounts of ATP; hence, a high density of mitochondria are needed to accommodate their high energy requirement. As such, these mitochondria, filled with reactive intermediates and pro-apoptotic signals, are intimately involved in I/R injury [60]. The inner mitochondrial membrane is responsible for maintaining mitochondrial transmembrane potential, and is usually impermeable to ions and proteins [61]. However, under stress, the opening of the mitochondrial permeability transition pore (mPTP) creates a non-selective channel between the inner membrane of the mitochondrion and the sarcoplasm. Consequently, a loss of the electrochemical gradient, production of ROS, Ca^2+^ overload, and formation of apoptosomes ensue [60].

Production of free radicals through partial reduction of oxygen during I/R is well understood. These highly reactive ROS can rapidly overwhelm the cell’s endogenous antioxidant self-defense system. This creates cellular injury by damaging lipids, proteins, DNA, and RNA. The xanthine oxidase substrates, xanthine and hypoxanthine, accumulate during ischemia, which triggers xanthine oxidase activation and consequently more ROS production [62]. These ROS can also elicit the opening of the mPTP resulting in a positive feedback loop of increased ROS production from the mitochondria (“ROS-induced ROS release”) [60,63]. A recapitulation of some of the underlying pathophysiological mechanisms is shown in Table 1 (modified according to [60]).

Many cellular and molecular processes contribute to ventricular remodeling in response to myocardial irradiation, myocardial I/R injury, hypertension, neurohumoral activation or other pathophysiological stimuli [64]. For example, excessive production of endothelin-1 (ET-1), angiotensin II, catecholamines and pro-inflammatory cytokines, which bind to their cognate receptors and activate the downstream signaling events, results in these pathological changes. This is then followed by necrosis, apoptosis, autophagy, or hypertrophy of the cardiomyocytes. It also induces fibroblast activation to produce collagen and other proteins that cause fibrosis [65,66,67]. 

Currently, restoring blood flow in an acutely occluded vessel represents the most effective, long-term clinical therapy for acute myocardial infarction [68]. Although restoration of blood flow is critical, the perfusion of oxygen-rich blood induces cytotoxic ROS production. This eruption of ROS leads to cellular necrosis and apoptosis, a process is referred to as “lethal reperfusion injury” [69]. Reperfusion injury accounts for up to 50% of the final size of the infarct [70]. Besides the cascade of events occurring within cardiomyocytes, the endothelium is also actively involved in the I/R injury. 

Nitric oxide (nitrogen monoxide, NO) is a signaling molecule involved in many physiological and pathological processes and a vasodilator in the vascular system. The endothelium is the major source of NO. Under healthy conditions, NO elicits vasodilation, which provides protective effects during I/R, in part by influencing oxygen consumption, platelet aggregation, leukocyte adhesion, and free radical metabolism [71,72,73]. In contrast to normal concentrations, high concentrations of NO exacerbate I/R injury largely by producing highly reactive peroxynitrite [74,75]. In addition to NO, the coronary endothelium has several other pathophysiological roles in I/R, such as serving as a source of non-NO vasoactive substances, activating the immune system, and an increased expression of cytokines, chemokines, and various adhesion molecules [60].

### 2.3. Heart Transplantation

I/R injury also occurs during cardiac transplantation, since such procedure requires cold preservation followed by warm reperfusion of cardiac grafts. The injury occurring during preservation or reperfusion can affect cardiac function after heart transplantation. Reducing injury is important for preserving cardiac function [76]. I/R injury is intimately associated with endothelial and parenchymal cell injury, increased vascular permeability, pathological inflammatory response, and the generation of toxic ROS. Modified preservation solutions including machine perfusion and other approaches have been developed to reduce the I/R injury [76]. Dysregulated redox balance during I/R is considered a primary driver of the injury as it causes oxidative damage and cellular aberrations. Additionally, mitochondrial dysfunction, neutrophil priming, xanthine oxidase, and NADPH oxidases all play a pivotal role in contributing to the redox dysregulation and the resultant I/R injury [77].

#### Antioxidants for Graft Preservation

In order to maintain the redox balance and thus improve the viability of the grafts and reduce the risk of post-transplant dysfunction, antioxidant treatments are often used [76]. Mitochondria, the major sources of ROS, are particularly susceptible to oxidative damage. To protect mitochondrial integrity during ischemia and reperfusion, several antioxidant molecules have been studied and investigated in preclinical and clinical studies. Melatonin, a well-known endogenous antioxidant, is also an inhibitor of inducible nitric oxide synthase (iNOS), and can enhance the expression of endothelial nitric oxide synthase (eNOS) [78,79]. Melatonin has been shown to mitigate I/R-induced mitochondrial swelling and protect liver grafts from I/R injury [80,81,82].

Ascorbic acid (AA) is a potent physiological scavenger of ROS, and is often added into Custodiol HTK and Polysol solution in order to prevent ROS-induced cell damage. However, high concentrations of AA can exacerbate hepatic I/R injury, possibly due to its redox interaction with iron [83].

α-Tocopherol (vitamin E), a lipid-soluble antioxidant, attenuates the propagation of lipid peroxidation in both cell membranes and plasma lipoproteins. Trolox is a water-soluble analogue of vitamin E with similar antioxidant properties. In a porcine heart transplantation model, Trolox-UW perfusion demonstrated significant therapeutic antioxidant effects against I/R injury [84]. However, a meta-analysis suggests that high-dose vitamin E (>400 IU/day) should be avoided, as it tends to increase all-cause mortality [85].

Another method has been used to inhibit enzymes involved in ROS production. For example, matrix metalloproteinases (MMPs) are associated with oxidative stress in cardiovascular diseases. The antibiotic doxycycline of the tetracycline family, has been found to inhibit MMP-2 expression and thus protects cardiac function from I/R injury [86], but its clinical use and effectiveness is limited. Similarly, inhibitors of mitochondrial respiratory complexes I and III prevent reperfusion by impairing ROS generation and thus improve cellular viability [87,88]. However, therapy to reduce free radicals during early reperfusion failed to relieve this pathological cascade of oxidative damage after reperfusion injury [89].

Whether combined utilization of several antioxidant approaches is more effective than simply reinforcing the antioxidant capacity of organs by a single chemotherapeutic agent remains to be seen. However, combined utilization of antioxidants, coupled with the stimulation of the antioxidant capacity of organs, might be effective, and is worth more intensive research. Unfortunately, the required therapeutic dosages of conventional antioxidants, or endogenous antioxidant inducers, often exhibit high toxicity at the needed levels, which obviously limits their usage to a narrow and ineffective range for the prevention of oxidative stress-related diseases.

## 3. New Approach for Prevention and Treatment of Cardiovascular Disorders

### 3.1. Medical Gases and Hydrogen on the Cardiovascular System

As mentioned previously, medical gases, specifically the three recognized gasotransmitters CO, H_2_S, and NO, have been shown to have significant cytoprotective benefits [51,90]. NO is perhaps the most well-recognized as a critical gaseous-signaling molecule that, under normal conditions, induces vasodilation, reduces superoxide production, decreases inflammation, and improves mitochondrial energy production. For example, ventilation of non-heart-beating donor lung grafts with NO during warm ischemia, ex vivo perfusion, and post-transplantation can reduce I/R lung injury [91].

CO possesses a high affinity for heme prosthetic group, and when supplemented in preservation solution, has also been demonstrated to improve the graft function in experimental studies [92,93]. Hydrogen sulfide (H_2_S) is considered as the third gaseous-signaling molecule that can induce relaxation of vascular smooth muscles, inhibit apoptosis, modulate inflammatory response, and also alleviate oxidative stress [94]. Although not considered a gasotransmitter, H_2_ is now considered a gaseous-signaling molecule with physiological and therapeutic benefits similar to that of nitric oxide (NO), carbon monoxide (CO), and H_2_S [95,96].

### 3.2. Molecular Hydrogen

Molecular hydrogen is the lightest gas and smallest molecule. It is often used in deep-sea diving, namely hydreliox (49% H_2_, 50% helium and 1% O_2_) to prevent decompression sickness [97]. One of the earliest therapeutic applications of H_2_ was published in the 1970s, wherein mice with skin-transplanted tumors were treated with hyperbaric hydrogen, which resulted in marked regression of the tumors [98]. Since hyperbaric H_2_ is not an auspiciously clinically viable option, H_2_ was put on the sidebar. However, it was later reported in 2007 that H_2_ treatment at clinically viable doses significantly reduced the levels of ^•^OH in cultured cells, and provided therapeutic neurological benefits [99]. This result suggested that hydrogen may potentially be an ideal antioxidant in clinics [100].

Hydrogen can be delivered via several methods, such as inhaling H_2_, drinking H_2_-rich water (HRW), injecting H_2_-rich saline (HRS), taking an H_2_ bath, dropping H_2_-rich saline into the eyes, and increasing the production of intestinal H_2_ by bacteria via non-digestible carbohydrates/certain medications [101,102,103,104,105]. Inhalation of H_2_ or the administration of HRW can increase the concentration of H_2_ in arterial and venous blood in proportion to the administered dose [106]. Inhalation of H_2_ gas leads to a plasma concentration that is in accordance with Henry’s law, which suggests that a 2% concentration of H_2_ results in approximately a 15.6 μM H_2_ concentration in the blood.

HRW can be prepared by dissolving H_2_ gas in water under high pressure or dissolving hydrogen-producing tablets in water [107]. The solubility of H_2_ is 1.56 mg/L at standard ambient temperature and pressure (SATP). Although 1.6 mg in 1 L solution initially appears insignificant, the number of “therapeutic moles” (i.e., H_2_) is greater than the number of “therapeutic moles” (i.e., vitamin C) in a 100 mg dose of vitamin C (i.e., 0.78 millimoles vs. 0.56 millimoles) due to the differences in molar mass [108]. Moreover, some H_2_-producing tablets can supersaturated the water, delivering more than five mg of H_2_ per tablet [107]. In some cases ingestion of HRW may show a more prominent effect than inhalation of H_2_ gas, even though the dose of hydrogen from water is lower than that of inhalation [10,109]. Ingestion of HRW reaches a peak in 5–15 min and returns to baseline levels 45–90 min after administration depending on the administered dose [110]. 

Molecular hydrogen has favorable physicochemical properties as a therapeutic antioxidant. It is electrically neutral and even smaller than molecular oxygen. As such it can easily penetrate cell membranes and diffuse into cellular organelles, such as the nucleus and mitochondria [111]. Additionally, the reactivity of H_2_ is so mild that H_2_ does not react with important physiologically relevant ROS that are involved in cell signaling. Hydrogen also has no effect on physiology, temperature, blood pressure, pH, or pO_2_ [112], and has not been reported to be toxic at concentrations even far above the clinically effective dosages. Excess H_2_ is simply expired via the lungs when too much is delivered [102].

During the past 12 years, basic and clinical research has demonstrated that H_2_ is an important biological regulatory factor with anti-oxidative, anti-inflammatory and anti-apoptotic effects on cells and organs [113]. Hydrogen attenuates the oxidative damage between hydroxyl radicals and biologically essential molecules. By reducing the levels of oxidized macromolecules, mitochondrial and cellular injuries are greatly reduced [114]. Hydrogen has been shown to reduce hyperbaric oxygen-induced toxicity to PC12 cells, while also maintaining the antioxidant levels of superoxide dismutase (SOD), catalase and glutathione peroxidase. These all contribute to hydrogens ability to alleviate the detrimental effects of hyperbaric oxygen [114].

It also was demonstrated that repeated inhalation of hydrogen-oxygen mixed gas [67%:33% (V/V)] significantly decreased both the acute and chronic stress-induced depressive- and anxiety-like behaviors of mice. Furthermore, ELISA analysis showed that it prevented the stress-induced increase in the serum levels of corticosterone, adrenocorticotropic hormone, interleukin (IL)-6, and tumor necrosis factor α (TNF-α) [115].

#### 3.2.1. Mechanisms of H_2_ Action

The biological effects of hydrogen have been attributed to major molecular mechanisms:

(1) specific scavenging activity of hydroxyl radicals and peroxynitrite
(2) reduction of inflammatory reactions
(3) modulation of signal transduction
(4) alterations of gene expressions

In addition to the direct effect of H_2_ on scavenging of ^•^OH, the reduction in inflammation may also be due to the mechanism of gene expression change. However, only the first mechanism of scavenging can be considered a primary target or direct mode of action. The other mechanisms are also not independent of each other, and influencing either one of the other mechanisms (e.g., signal transduction) can influence a different mechanism (i.e., gene expression). Moreover, scavenging of reactive chemical species could also be responsible for regulating and driving the other proposed mechanisms of modulating signal transduction and altering gene expression. For example, peroxynitrite can regulate gene expression through the nitration of various proteins involved in transcriptional regulation [116]. Ingestion of hydrogen water suppresses the nitration of proteins; thus, it is possible that a small amount of H_2_ gas ingested by drinking HRW can influence nitration in vivo and result in alterations of gene expression [1,106,117].

Catalase and SOD are extremely efficient at detoxifying H_2_O_2_ and O_2_^•−^, respectively. These enzymes are critical in mitigating the ROS-induced cellular injury. By regulating their concentration, they also prevent the production of hydroxyl radicals as they can be converted to ^•^OH radicals via the Haber-Weiss and Fenton reaction in the presence of catalytically active metals such as Fe^2+^ and Cu^+^ [32,118] (see also Figure 1). Hypothetically, administration of these enzymes could be very beneficial, especially to the vascular endothelium suffering from oxidative injury [119]. However, due to their inability to cross cell membrane barriers coupled with their fast elimination, their clinical translation is thwarted by an insufficient delivery of these enzymes to the desired sites.

In contrast, H_2_ can rapidly diffuse through the cell membranes and lipid bilayers, reaching the nucleus and mitochondria where most pugnacious ROS are located. The direct scavenging of the hydroxyl radical according to the exothermic reaction of H_2_ + ^•^OH → H_2_O + H^•^ followed by H^•^ + O_2_^−^ → HO_2_^−^ reaction was postulated as a potential mode of action decades earlier [98]. This would be highly desirable since the ^•^OH reacts nearly instantaneously with cellular biomolecules and has been postulated to be the primary initiator of oxidative injury. Unlike other reactive oxygen species, there is no known enzyme specifically equipped to handle the ^•^OH radical, likely due to its non-selective rapid reaction with the nearest nucleophilic biomolecule. Therefore, the ability for H_2_ gas to selectively react and neutralize the hydroxyl radical is a highly desirable property. 

Many of the harmful consequences of disease, irradiation, I/R injury and other assaults can be attributed to the hydroxyl radical; it is, therefore, logical to conclude that the mechanism of hydroxyl radical scavenging by hydrogen is correct, since the administration of molecular hydrogen seems to alleviate the same harmful damages attributed to the hydroxyl radical. Moreover, the direct scavenging of the hydroxyl radical is currently the only primary direct mode of action that has been postulated, and, as discussed, scavenging of toxic reactive molecules can in turn result in changes in both signal transduction and gene expression. However, although the mechanism of direct scavenging by hydrogen is the only one considered to be a primary/direct mode of action, unfortunately it cannot fully explain all the diverse biological effects of H_2_ [7]. Moreover, the biological significance of this reaction is debated [108,120,121]. This is partly because the 2nd-order reaction rate constant between hydroxyl radicals and H_2_ (4.2 × 10^7^ M^−1^ s^−1^) is about three orders of magnitude lower than that between other more abundant nucleophilic cellular components [108]. Additionally, the scavenging of the hydroxyl radical would not only produce the inert byproduct of water but also either the highly reactive atomic hydrogen radical or the even more reactive solvated electron (H_2_ + ^•^OH → H_3_O^+^ + e^−^), each of which could induce oxidative damage as would the hydroxyl radical [122]. 

Moreover, H_2_ is only transiently present in the body, yet its biological and antioxidant effects remain well-after H_2_ has been cleared from the body [123]. This may suggest that the mechanism may have more to do with signal modulation than direct radical scavenging [108,110]. H_2_ seems to modulate the expression of diverse genes, including NF-κB, c-Jun N-terminal kinase (JNK), fibroblast growth factor 21 (FGF-21) [124], peroxisome proliferator-activated receptor-γ coactivator-1α (PGC-1α) [125], proliferation cell nuclear antigen, vascular endothelial growth factor (VEGF), glial fibrillary acidic protein (GFAP), and many other transcription factors and regulatory proteins [10,126]. However, these molecules are likely downstream or indirectly regulated by H_2_, as the direct targets of H_2_ have yet to be elucidated [10].

Taken together, it is clear that although the selective extinctions of hydroxyl radical and peroxynitrite were initially proposed as underlying mechanisms, there must be other explanations [127]. Molecular hydrogen regulates signaling pathways and gene expression via modulating the expression/activities of various biomolecules, as well as several miRNAs, which may account for the therapeutic effects of anti-reperfusion injury, anti-radiation injury, anti-inflammation, anti-apoptosis, anti-metabolic disorders, anti-allergy, anti-dementia as well as anti-aging [7,10]. 

#### 3.2.2. H_2_ Modulates Nrf2 Pathway

**Figure 2:**
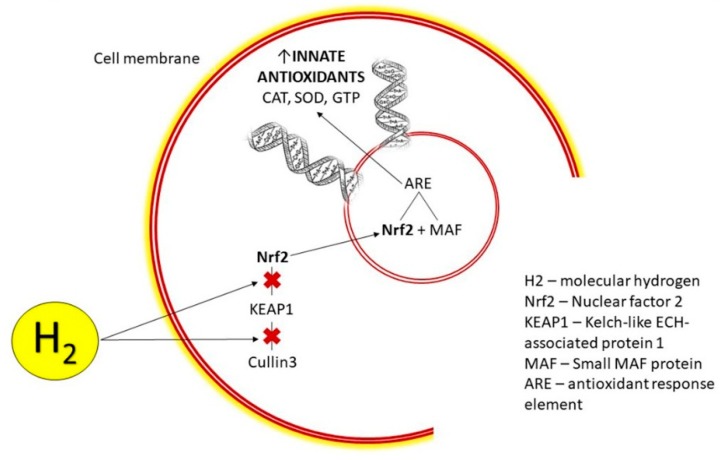
Mechanism of H_2_ action: transcription and production of innate antioxidants upon entry into cell cytoplasm, release and accumulation of Nrf2 and its translocation into the nucleus. CAT = catalase, SOD = superoxide dismutase, GTP = glutathione peroxidase.

A significant mechanism in the cellular defense against oxidative stress is induction of phase II enzymes via activating the Nrf2-antioxidant response element (ARE) signaling pathway (Figure 2). The nuclear factor erythroid 2-related factor 2 (Nrf2) is considered an important regulator of electrophile/antioxidant homeostasis, and supports the functional integrity of cells, particularly under conditions of oxidative stress [8]. This pathway regulates the expression of over 200 genes involved in antioxidation and detoxification. A dysregulated cellular redox status due to elevated levels of ROS and/or a reduced antioxidant status is an important signal for inducing the transcriptional response mediated by this enhancer protein [128].

Under unstressed conditions, Nrf2 levels are regulated in the cytoplasm by the Kelch-like ECH-associated protein 1 (Keap1 protein), which prevents its release into the nucleus and promotes its degradation [129]. The rate of Nrf2 ubiquitination and its degradation in non-stressed cells appears to be largely dependent on the concentration of the Keap1 protein [129]. Keap1 is also the sensor for a wide array of small molecule activators/inducers of Nrf2 [130].

Activation of the Nrf2 pathway in response to stress signals induces the dissociation of Nrf2 from the Keap1 protein, which allows for the Nrf2-transcription factor to translocate into the nucleus, where it binds to the cognate DNA regulatory element termed ARE or electrophile-responsive element (EpRE) [128,129,131]. The binding initiates the transcription of antioxidative genes resulting in the production of many cytoprotective proteins [132].

Molecular hydrogen has been demonstrated to activate the Nrf2/EpRE signaling pathway [133], which has previously been reviewed [10]. Our group demonstrated that, in vivo, H_2_ activated the Nrf2 pathway, which resulted in the prevention of irradiation-induced lipid peroxidation of the rat heart [8]. H_2_ administration for 9 days significantly elevated SOD-2, and increased the phosphorylation of Akt kinase at Ser473, a cell-survival signaling molecule involved in the regulation of Nrf2 [8,134]. It is likely that many of the therapeutic effects of H_2_ may be attributed to the activation of the Nrf2 pathway, which stimulates the production of innate antioxidants as well as the reduction of apoptosis and inflammation [8].

#### 3.2.3. H_2_ Induces Hormesis

The activation of the Nrf2 pathway by H_2_ may appear somewhat paradoxical since the H_2_ molecule is considered to be a reducing agent and the Nrf2 protein seems to be induced by electrophilic chemical species and suppressed by mild nucleophilic substances [131]. Indeed, oxidative stress is the major activator of the Nrf2 pathway. For example, the lipid peroxidation product, 4-hydroxy-2-nonenal, in cardiomyocytes mediates the Nrf2-dependent upregulation of uncoupling protein 3 (UCP3) [135]. This effect might be particularly important in mediating the protective effects of pre-conditioning, which induces mild oxidative stress and subsequent upregulation of various proteins including cytokines, heat-shock proteins, NF-κB, and Nrf2 [8,136,137,138]. This process of a stress being mildly toxic followed by increased cellular protection is referred to as hormesis [139]. 

Hydrogen has been shown to mimic the effects of a mild hormetic stress, for example by transiently increasing superoxide production [140], slightly increasing markers of oxidative stress, MDA and 8-OHdG [110,141], activating NF-κB [142,143], and inducing heat shock proteins [144] and the mitochondrial unfolded protein response [145]. The hormetic actions of molecular hydrogen have recently been compared to those of regular exercise. LeBaron et al. proposed that hydrogen may act as an exercise mimetic and redox adaptogen via mild hormetic mechanisms [108,140]. 

#### 3.2.4. H_2_ and miRNAs 

Another emerging mediator of hydrogen’s biological effects are miRNAs. These non-coding RNAs, because of their imperfect pairing with target messenger RNAs, modulate the mRNA stability and/or their translational efficiency. They are considered novel regulators of oxidative and inflammatory stress that modulate the expression of multiple redox-related genes. The miRNA-200 family members regulate oxidative-stress dependent endothelial dysfunction in cardiovascular complications. Other miRNAs, such as miRNA-210, are involved in mitochondrial metabolism [146]. Because miRNAs modulate a diverse spectrum of cardiac function with developmental, pathophysiological, and clinical implications [3], miRNAs show different expression profiles in the normal and diseased heart. This may allow for differences in miRNA expression to serve as a viable diagnostic marker of heart disease [147,148]. It is suggested that a specific class of heart disease can be predicted with an accuracy probability of 69% by using miRNA expression patterns [1]. This also suggests the miRNAs may serve as potential therapeutic targets for various conditions [149].

ROS-induced aberrations in miRNA levels can lead to carcinogenesis via activating various oncogenes or the silencing of tumor suppressor genes [150]. An understanding of the exact mechanism of how miRNAs influence the effectors of ROS production and redox signaling pathways in cells of the cardiovascular system requires additional research [151].

Irradiation induces oxidative stress and pathologically influences the expression of several miRNAs including miRNA-1 and miRNA-21. An upregulation of miRNA-21 expression is associated with myocardial hypertrophy and fibrosis [3,152,153]. It has also been correlated with an increased expression of protein kinase C [154], which is also implicated in tissue remodeling. MiRNA-21 has been reported as a novel promising target in cancer radiation therapy [29,155]. 

Our experiments demonstrate that molecular hydrogen can attenuate the irradiated-induced aberrant miRNA expressions in rats including, miRNA-1, miRNA-9, miRNA-15b and miRNA-21, and miRNA-199 [1,141,149,156]. 

Furthermore, analysis of miRNA profiles of hippocampal neurons during I/R injury revealed that hydrogen inhibits I/R-induced expression of the miRNA-200 family by reducing ROS production, which has led to suppression of cell death [156]. 

The underlying mechanisms explaining how hydrogen modulates these miRNA expression, and to what extent their expression is due to the downstream or upstream effect of H_2_, remain to be elucidated.

## 4. Potential Usage of H_2_ Against Diseases

### 4.1. Therapeutic and Protective Function of H_2_ in Chemotherapy and Radiotherapy 

Chemotherapy and radiotherapy are associated with increased oxidative stress, which further induces pathological cellular aberrations [157,158]. H_2_ may be considered superior to some antioxidants since unlike conventional antioxidant supplements, hydrogen cannot neutralize important reactive oxygen and nitrogen species that are involved in biological signaling [99], and it can easily diffuse throughout the body, tissues, organs, and cells without affecting signaling reactive species [99,159].

H_2_ also reduces oxidative stress, inflammation, and apoptosis by regulating gene expression [1]. Emerging evidence has demonstrated the pleiotropic therapeutic effects of molecular hydrogen in a variety of animal disease models as well as in many human diseases [11,113,160]. For example, H_2_ has been demonstrated to reduce the expression of several pro-inflammatory mediators and markers of oxidative stress and apoptosis including TNF-α, IL-6, IL-1β, IL-10, IL-12, chemokine ligand 2 (CCL2), intercellular adhesion molecule 1, NF-κB, nuclear factor of activated T-cells (NFAT), high mobility group box 1 protein, prostaglandin E2, cyclooxygenase-2 (COX2), serum diamine oxidase, tissue MDA, protein carbonyl, TBARs, myeloperoxidase activity, JNK, and caspase-3 bringing there levels within or preventing their levels from diverging away from normal the homeostatic range [161,162,163].

**Figure 3:**
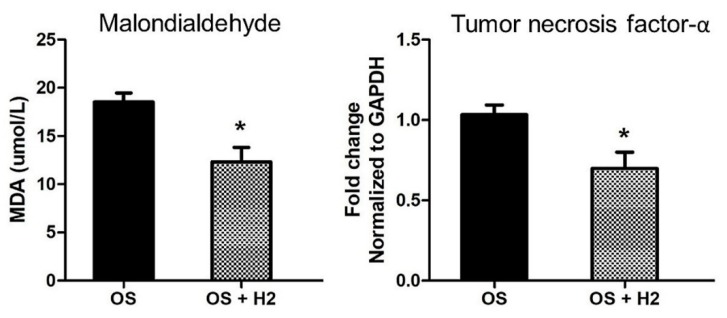
Effect of molecular hydrogen on irradiation-induced lipid peroxidation and inflammation. The marker of oxidative stress, malondialdehyde (MDA), was elevated in blood plasma after irradiation of rat myocardium. Application of molecular hydrogen (H_2_) significantly decreased the levels of MDA. Myocardium irradiation increased levels of the inflammatory marker TNF-α in the rat´s heart tissue. Significant reduction of TNF-α was observed after H_2_ treatment. OS = oxidative stress, H_2_ = molecular hydrogen. Values are means ± SD, *n* = 5, *: *p* < 0.05. Modified from reference [8].

These therapeutic effects of molecular hydrogen are important in mediating the cytoprotection against radiotherapy and chemotherapy. Hydrogen has been shown to exert radioprotective effects on cultured cells and mice [25,164]. For example, in irradiated animals, the lipid peroxidation marker MDA was significantly increased in the small intestine, but was not similarly elevated in the hydrogen-water group [165]. Our group demonstrated a similar effect of hydrogen on the myocardium by pretreating rats with HRW prior to myocardial irradiation [9]. H_2_ attenuated the markers of radiation-induced inflammation (i.e., TNF-α), lipid peroxidation (i.e., MDA), and also the pathological changes in miRNAs [141], which was corroborated by another group (Figure 3) [166].

Similarly, the antioxidant function of HRS was investigated in a rat model of radiation toxicity. Intraperitoneal injection of HRS before radiation protected the gastrointestinal endothelia from radiation-induced injury, decreased plasma MDA and intestinal 8-OHdG levels, and maintained plasma levels of endogenous antioxidant enzymes including SOD and glutathione-S-transferase [25,167]. Subcutaneous injection of HRS before irradiation markedly reduced the severity of radiodermatitis, and accelerated tissue recovery [168]. 

In addition to hydrogen administration in water or saline, hydrogen-containing gas (HCG) (1.3% hydrogen + 20.8% oxygen + 77.9% nitrogen) is also a viable option. HCG coincides with the many requirements of an ideal radioprotectant, such as efficacy, broad spectrum, acceptable administration and little to no toxicity [28]. HCG was used on a rat model of radiation-induced dermatitis and on healing-impaired skin wounds [169]. The study found that pre-inhalation of HCG effectively alleviated the severity of acute radiodermatitis and stimulated the healing of radiation-induced skin injury by reducing cytotoxic ROS and preventing the radiation-induced apoptosis of epidermal keratinocytes (EKCs) with no toxic effects [169]. Thus, the inhalation of HCG may be an easy and safe pre-treatment to prevent the dermatitis. HCG pre-treatment may serve as a new clinical therapy in the treatment of radiodermatitis and oxidative damage caused by radiation treatment.

The many animal studies on H_2_ lend support for the use of molecular hydrogen in clinical practice. Many side effects of radiotherapy appear to result from the increased oxidative stress and inflammation generated during radiotherapy. In addition to the heart, lungs and skin, the gastrointestinal tract is also very susceptible to radiation damage. As low as 1 Gy of radiation induces a dramatic increase in apoptosis in mouse small intestinal crypt within three to six hours after exposure [170]. Consequently, radiation treatment in cancer patients often results in fatigue, induces gastrointestinal (GI) toxicity, and impairs quality of life. 

The ingestion of HRW in a small clinical study of 49 patients receiving radiotherapy for malignant liver tumors was investigated in a randomized, placebo-controlled trial. HRW was prepared by metallic magnesium [Mg + 2H_2_O → Mg(OH)_2_ + H_2_] at an H_2_ concentration of 0.55–0.65 mM. Results showed that daily consumption of HRW for 6 weeks reduced reactive oxygen metabolites in the blood and maintained blood oxidation potential of these patients. Moreover, the quality of life during radiotherapy was improved in patients drinking HRW compared to placebo. Importantly, the HRW did impair the anti-tumor effect of radiotherapy [171], which has been a concern, although debated due to equivocal findings with conventional antioxidants [172]. 

Additionally, molecular hydrogen may be useful to combat the harmful effects of other anti-tumor and chemotherapy drugs. For example, one group reported that inhalation of 1% H_2_ gas or drinking H_2_ water attenuated the cisplatin-induced nephrotoxicity, mortality, and loss of body weight. Drinking H_2_ water also decreased the level of apoptosis in the kidney. Similar to the protective effects of radiotherapy, H_2_ did not compromise the antitumor effects of cisplatin either in cancerous cell lines *in vitro* or in tumor-bearing mice in vivo [173]. 

Taken together, these results suggest that both inhalation of H_2_ gas and oral administration of H_2_ water may protect against inflammation and oxidative-stress related cancer drugs, and thus improve the antitumor effect in the clinical management of cancer [165].

### 4.2. H_2_ Benefits in I/R Injury

The favorable chemical, physical and biological properties of H_2_ qualify it as an excellent candidate for the prevention and treatment of I/R injury. Gut microbiota-derived H_2_ slightly, but significantly, reduces myocardial infarct size [174]. Inhaled H_2_ is rapidly transported to the ischemic area before coronary blood flow is reestablished in the occluded region [64]. The 2007-seminal paper on hydrogen [99] demonstrated that H_2_ gas decreased cerebral infarction induced by cerebral artery occlusion. Shortly thereafter, it was reported that inhalation of H_2_ gas during reperfusion of the liver, following 90 min of blood-flow occlusion, significantly suppressed hepatic cell death and injury, whereas helium gas showed no protective effects [175]. In a rat model of myocardial I/R injury, inhalation of 2% H_2_ at the onset of ischemia, and for 60 min after reperfusion, reduced infarct size, lowered LV-end-diastolic pressure, attenuated pathological remodeling, and improved cardiac function 30 days after the myocardial I/R injury [104]. In swine, inhalation of 4% H_2_ improved myocardial stunning, and reduced myocardial infarct size [104,176]. Similarly, inhalation of 2.4% H_2_ gas during and for 24 h after cardiopulmonary bypass in swine significantly attenuated the neurological and renal injury induced by the ischemic assault [177]. 

Although nitric oxide also has the ability to decrease the infarct size in myocardial I/R injury [176,178], it also has toxic effects largely attributed to its production of various reactive nitrogen species, specifically peroxynitrite. Peroxynitrite is a perniciously reactive molecule that reacts with the tyrosine at the active site of essential enzymes (such as Tyr6, Tyr32, and Tyr78 in mouse GST-μ) and other cellular components [176,179]. Propitiously, these adverse effects can be reversed by H_2_ inhalation [174]. Breathing NO with H_2_ can decrease cardiac injury and enhance recovery of the left ventricular function, by abolishing the toxic byproducts of NO metabolism [174].

In addition to H_2_ inhalation, intraperitoneal injection of H_2_-rich saline attenuates myocardial I/R injury and improves cardiac function through its anti-oxidative, anti-apoptotic and anti-inflammatory effects [147,180]. Similarly, hydrogen-rich saline injection prior to reperfusion significantly reduced the level of myocardial 8-OHdG and MDA in the area-at-risk zones [147]. I/R leads to rapid calcium accumulation and ROS production, which triggers the opening of the mitochondrial permeability-transition pore [181]. This results in a loss of the membrane potential and induction of apoptotic signaling. It appears that at the onset of reperfusion, H_2_ is able to reduce ROS generation and thus reduce DNA damage and lipid peroxidation, while also preserving mitochondrial membrane potential and ATP synthesis [61]. These all work together to protect the heart by inhibiting the opening of the mitochondrial permeability-transition pore [147]. Importantly, HRS administered only 5 min before reperfusion was enough to exert these protective effects, including the inhibition of caspase-3 signaling activation, which consequently reduced cardiomyocyte apoptosis [147]. 

Besides the I/R injury, excessive neurohormonal activation can similarly induce cardiac complications such as those from isoproterenol (ISO), a β-adrenoreceptor agonist. Promisingly, intraperitoneal injection of H_2_, 7 days prior to and 7 days with ISO administration, protected against ISO-induced cardiac hypertrophy and dysfunction in vivo, and H_2_-rich medium attenuated ISO-induced cardiomyocyte hypertrophy *in vitro* [105]. Similarly, HRS protected against high-dose ISO-induced acute myocardial infarction in a rat model by its anti-oxidative and anti-inflammatory activities [182]. H_2_ suppressed ISO-induced excessive cardiomyocyte autophagy both in vivo using a mouse model and *in vitro* using H9c2 cardiomyocytes in H_2_-rich media [183]. 

Correspondingly, 2% inhalation of H_2_ mitigated myocardial I/R injury in rats by reducing cardiac endoplasmic reticulum stress and autophagy [184]. HRS also protected against doxorubicin-induced rat myocardial injury [185] and improved survival and neurological outcomes after cardiac arrest/resuscitation in rats [186]. H_2_ inhalation also improved survival and functional outcomes in a rat model of post-cardiac arrest syndrome [187]. The protective effects of H_2_ on cardiac hypertrophy were also confirmed in a mouse model of spontaneous hypertensive rats. HRS administration effectively attenuated left ventricular hypertrophy via suppression of inflammation and oxidative stress, maintaining mitochondrial function, and suppressing angiotensin II levels locally in left ventricles by downregulating angiotensin-converting enzyme [188].

**Figure 4:**
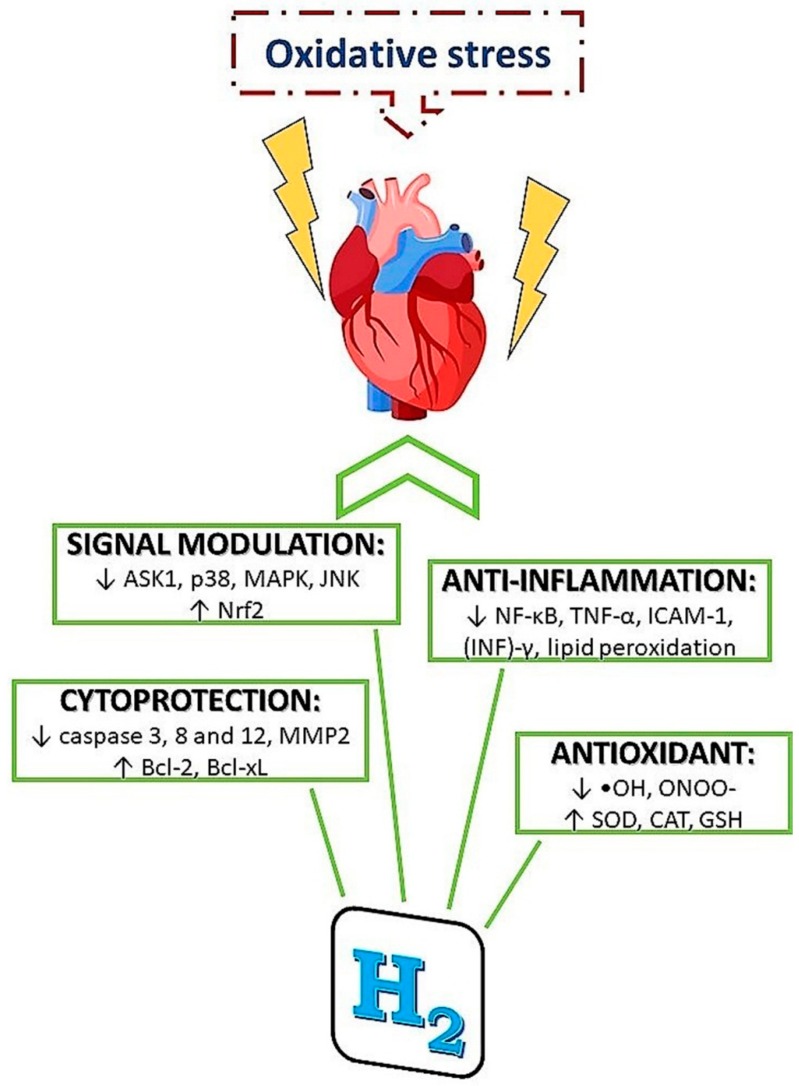
Mechanisms of molecular hydrogen action in condition of increased oxidative stress. Molecular hydrogen has been demonstrated to provide protective effects via several mechanisms including antioxidant, anti-inflammatory and cytoprotective actions, as well as via signal modulation.

H_2_ appears to provide some of its protective effects by decreasing the NADPH oxidase expression and preventing mitochondrial damage. These effects lead to the decrease of ROS accumulation, which subsequently modulates downstream ERK1/2, p38, and JNK signaling pathways [64,189]. Figure 4 illustrates some of the mechanistic protective effects of H_2_.

### 4.3. H_2_ for Graft Preservation

H_2_ administered to excised cardiac grafts during cold preservation significantly reduced cold-induced I/R injury in grafts from syngeneic older donors and in allografts subjected to extended cold storage [190]. Application of hydrogen likely has beneficial effects in conditions of hypoxic post-conditioning, which strengthen its cardioprotective efficiency [3]. Zálešák et al. [138] reported that H_2_-saturated Krebs–Henseleit solution significantly decreased infarct size induced by myocardial I/R hypoxic post-conditioning [138]. 

Hydrogen-rich preservation solution has been tested in liver, kidney, pancreas, bone marrow, lung, and intestinal cold storage [191]. Studies demonstrate that these hydrogen-rich solutions have the ability to inhibit oxidative stress, suppress immune and inflammatory responses, inhibit release of high mobility group box 1 (HMGB1), improve mitochondrial function and energy metabolism, enhance graft survival, and attenuate cardiac injury during preservation or reperfusion in heart transplantation [190,192,193,194,195]. 

Taken together, it is hypothesized that the donor could undergo hydrogen inhalation, followed by storing the tissue in a hydrogen-rich preservation solution. This may be a promising and simple method to prolong the graft preservation time and the survival of recipient. Clinical investigation of this hypothesis is warranted and encouraged.

The timing of H_2_ may also be an important factor, which requires more research. For example, H_2_ was able to prevent ISO-induced cardiac dysfunction when administered several days prior, but not the day of ISO administration [64]. Therefore, pre-treatment of H_2_ may be more effective than post-treatment. Most studies seem to support that a pre-exposure to hydrogen is the most effective. For example, 2% hydrogen inhalation 1 h prior to liver procurement can also protect the liver from I/R injury by activating the NF-κB signaling pathway in the early phase followed by its subsequent downregulation [142].

Previously, other therapeutic strategies for combating ROS-induced cellular damage were speciously auspicious in animal models, but have mostly failed in human clinical trials [196]. In contrast, molecular hydrogen may prove to be a simple, safe, economical and novel approach for future cardiac protection [106,147]. Indeed one promising clinical study demonstrated that H_2_ inhalation (1.3% H_2_) during primary percutaneous coronary intervention (PCI) is a feasible and safe treatment option for patients with ST-elevated myocardial infarction, and may prevent adverse left ventricular remodeling after primary PCI [197].

## 5. Conclusions

Excessive production of free radicals is a mediator of many cardiovascular disorders like ischemia/reperfusion injury, including those related to grafts-storage during heart transplantation, as well as radiation-induced heart disease. To prevent, or at least mitigate, the development of oxidative stress, it is necessary to develop new approaches for effective and safe reduction of the pathological consequences of excessive ROS levels.

Studies have shown that H_2_ can treat many diseases associated with oxidative stress including cardiovascular disorders. Currently, it is suggested that the main mechanisms of H_2_ action are its modulation of signal transduction, alteration of gene expression, and, although debatable and needs further analysis into is biological significance, its selective ^•^OH-radical scavenging effect. However, the primary target(s) of molecular hydrogen remain elusive, but may involve the principle of hormesis. The greatest advantages for using H_2_ are easy penetration through all biological membranes, wide spectrum of administration form, and little or no significant reported adverse effects. Thus H_2_ may represent a novel therapeutic strategy to mitigate oxidative stress and its pathological consequences.

However, further research is still needed to determine the optimal method of administration, optimal dose and frequency, and the actual clinical impact of using H_2_ along with the potential risks/pitfalls connected with its administration.

## Figures and Tables

**Table 1:** Various mechanism underlying the pathophysiology of myocardial ischemia/reperfusion injury (modified according to reference [60]) JNK = c-Jun N-terminal kinase, AMPK = AMP-activated protein kinase, HIF-1α = Hypoxia-inducible factor 1-alpha.

Alteration Caused by I/R Injury	Mechanism
Changes in ion flux	Accumulation of intracellular calcium Ca^2+^-induced “stone-heart”
	Increased sodium influx
	Abnormal potassium flux
	Drop in pH followed by normalization upon reperfusion
Loss of mitochondrial membrane potential	Opening of mitochondrial permeability transition pore (mPTP)
	Cytochrome c release
	Reduction of ATP synthesis
Reactive oxygen species (ROS)	Substrate-level induction of xanthine oxidase resulting in more ROS
	Impaired mitochondrial function
	Neutrophil infiltration
	ROS-induced ROS
Dysregulated nitric oxide (NO) metabolism	Loss of NO-vasodilation
	Production of peroxynitrite
	Abnormal S-nitrosation
Apoptosis	JNK pathway
	Ceramide generation
	Cytoplasm acidification
	Caspase activation
Autophagic cell death	Excessive AMPK activation
	Excessive induction of HIF-1α
Endothelial dysfunction	Cytokine, myokine, chemokine signaling
	Expression of cellular adhesion markers
	Impaired vasodilation
Platelet aggregation	
Immune activation	Innate immunity (e.g., complement activation, induction of Toll-like receptors)
	Neutrophil accumulation
